# Economic and social determinants of life expectancy in China

**DOI:** 10.3389/fpubh.2025.1554336

**Published:** 2025-05-30

**Authors:** Ruitao Li, Yunqiu Zhan, Yinying Tao, Rong Wang, Xiao Gu, Zhenhua He, Bingcheng Zhu

**Affiliations:** ^1^Technology, Education, and Management at the Graduate School of Business & Advanced Technology Management, Assumption University of Thailand, Bangkok, Thailand; ^2^Intelligent Transportation School, Yunnan Vocational College of Transportation, Kunming, China; ^3^School of Marxism, Chengdu Technological University, Chengdu, China; ^4^School of Design and Fashion, Zhejiang University of Science and Technology, Hangzhou, China; ^5^School of Marxism, Yulin Normal University, Yulin, China; ^6^Regional Rural Governance Institute, Hangzhou Open University, Hangzhou, China; ^7^College of Medicine and Health Sciences, Shaoxing University Yuanpei College, Shaoxing, China; ^8^School of Statistics, Dongbei University of Finance and Economics, Dalian, China

**Keywords:** economic, social, life expectancy, China, ARDL

## Abstract

Life expectancy (LEX) is closely linked to SDG 3: Good Health and Wellbeing, which aims to ensure healthy lives and promote wellbeing for all ages. Improving LEX reflects progress in reducing preventable diseases, enhancing healthcare access, and addressing social determinants of health. By focusing on economic growth, education, and healthcare, countries can achieve sustainable improvements in life expectancy, contributing to the broader goals of SDG 3. Previous studies take economic or social determinants of LEX individually. Thus, this study fills the gap to empirically examines the impact of Economic and social determinants of LEX in China from 2000 to 2023. This study utilizes the Autoregressive Distributed Lag (ARDL), Fully Modified Ordinary Least Squares (FMOLS), and Dynamic Ordinary Least Squares (DOLS) estimators. The finding shows that health expenditure (HE), birth rate (BR), and education (EDU) have a positive effect on LEX. On the other hand, inflation (INF), population growth (PG), and mortality rate (MR) negatively affect LEX. This study has several policy recommendations based on findings to enhance the LEX in China.

## 1 Introduction

Health represents a critical component in the growth and development of society, encapsulating the fundamental aspects of economic, social, and sustainability for humanity on earth. Health is the foundation for developing a productive labor force necessary for societies' sustained progress and expansion (1). One key benefit of a healthier population is a more productive workforce, as healthy infants and children are better able to develop and contribute effectively in adulthood (2). According to the United Nations, the mortality rate for children under the age of five was alarmingly high, with ~5.4 million deaths recorded in 2017 (3). A nation's life expectancy (LEX) reflects its healthcare system's development and is a broader indicator of its overall health status (4). LEX at birth and postnatal mortality are pivotal criteria that shape the definition of health and inspire efforts to lead healthier lives (5). LEX has emerged as a key indicator of a nation's health status and is widely recognized as one of the most effective tools for measuring a country's development (6). LEX at birth refers explicitly to the estimated number of years a newborn is expected to live, assuming that current mortality patterns remain constant throughout their lifetime (7). This measure incorporates mortality rates across all stages of life, providing insights into a population's average mortality trends (8). Many economies around the globe grapple with financial inequality, inefficient resource utilization, and an over-dependence on non-renewable energy and human resources, all of which negatively impact LEX (8, 9). Life expectancy is a cornerstone of the Human Development Index, and efforts to extend it have been a significant focus of medical research (10). It plays a vital role in planning for population health, social services, and old-age pensions. Reliable forecasts of life expectancy for future periods enable governments to direct financial planning and policy development more effectively (11).

There are many economic determinants of LEX, such as economic growth (EG), inflation (INF), health expenditure (HE) etc. GDP per capita is among the most influential factors in improving life expectancy. Increased economic development is associated with longer lifespans, as demonstrated in countries like Spain and Italy, where rising economic activity is both a cause and an effect of extended life expectancy (5). Health improvements contribute to economic growth through three primary pathways: enhanced labor productivity, increased investment in physical and human capital, and reduced mortality and fertility rates (12, 13). Greater longevity and improved health are closely linked to increased productivity, essential drivers of sustainable economic expansion (10). As highlighted by Monsef and Mehrjardi (14), inflation is a significant economic determinant of LEX. In developing economies, rising and persistently high inflation disproportionately impacts vulnerable groups, such as children and women, increasing their risk of poor health and malnutrition. Inflation exacerbates these challenges by reducing affordability and negatively affecting wellbeing through multiple pathways. These include diminished investment in education, reduced consumption levels, malnutrition, and deteriorating health outcomes (3).

Healthcare spending is critical in improving health outcomes, including LEX, death rates, and overall mortality (15). Both government and private expenditures on health are considered investments in health capital, as they directly contribute to the health production system (13, 16). A significant portion of health expenditure (HE) is funded through public sources, including taxes and mandatory social insurance contributions. While some argue that public financing increases HE by reducing the net cost of care and stimulating demand, others contend that it may act as a limiting factor (62, 63). Both public and private HE are essential in ensuring access to health services. Utilizing these services and products produces positive health outcomes (13). From an economic perspective, public spending on healthcare should prioritize maximum efficiency while achieving the expected levels of effectiveness in outcomes such as surgeries, specialized medical care, and preventive medicine. Healthcare outcomes, influenced by behavioral, social, and other factors, are closely tied to life expectancy (17).

There are many social determinants of LEX, such as education (EDU), population, birth rate, etc. Education is widely recognized as a critical component influencing health and overall wellbeing, with considerable implications for life expectancy across varied groups (18). Numerous studies that have examined the relationship between education and health outcomes have found a substantial correlation between educational attainment and several health measures, including overall life expectancy, disease prevalence, and mortality rates (19). Higher education levels are often associated with better access to healthcare services, healthier lifestyles, and increased knowledge of preventative health practices, all of which increase life expectancy (18). Enhancing education gives people a tool that directly and indirectly lengthens their life expectancy (20, 64). People with less education have higher body mass indices and are more likely to be obese. Conversely, higher levels of education are linked to better diets and more frequent exercise (21–23). Education has an indirect impact on health and LEX by raising the risk of non-fatal workplace injuries and affecting employment in occupational classes with better salaries and access to health insurance, albeit the causal relationship is complex (19, 24–27). Higher levels of education typically result in better employment prospects with more excellent pay, which in turn enable people to possess more significant access to first-rate healthcare services and higher living standards. By increasing the effectiveness of health production, educational attainment can also increase life expectancy (28, 29). The population growth rate refers to the increase in a country, state, or city's population over time (30). Rapid population growth has been a source of contention, with some arguing that it will have a negative impact on citizens' health and life expectancy due to increased demand on healthcare facilities, while others argue that it will lead to increased productivity through innovation, production, or economies of scale in medical-care services (31–33). Life expectancy increases in emerging states are impeded by rapid population growth because of inadequate infrastructure and resource allocation (45). There has been a continuous and diverse discussion in many countries over the correlation between LEX and population growth rates (34). However, since the relationship between population increase and LEX differs from nation to nation, there is no universal agreement on whether it is good or bad (35). The availability and reliable supply of electricity play a crucial role in both the social and economic wellbeing of individuals and society. Studies have shown that access to electricity significantly enhances quality of life and contributes to higher LEX (2, 36).

**Figure A1:**
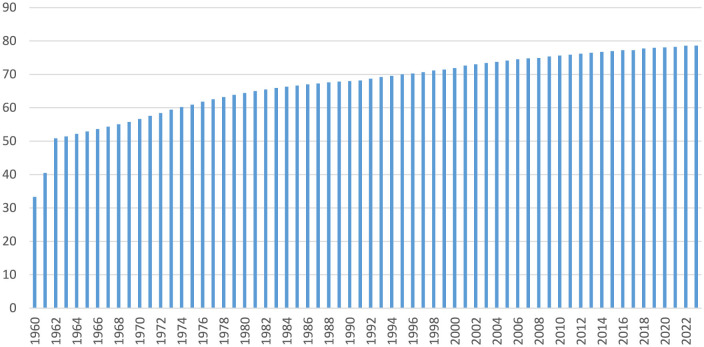
Trends of Life expectancy at birth, total (from 1960 to 2023). *Source* Authors own based on WDI data.

China's LEX has significantly risen over the past six decades, reflecting remarkable progress in healthcare, economic development, and living standards. The data from 1960 to 2023 shows a dramatic increase in LEX at birth, highlighting the country's advancements in medical care, disease control, and overall wellbeing (See, Figure A1). From 1960 to 1970, LEX experienced a rapid increase, rising from 33.275 years to 56.607 years. This sharp improvement suggests significant advancements in healthcare, nutrition, and living standards. Between 1970 and 2000, the growth continued steadily, with life expectancy increasing from 56.6 years to 71.9 years, likely driven by developments in medical technology, disease control, and public health policies. From 2000 to 2023, LEX rose slower but consistently, reaching 78.6 years. This steady growth can be attributed to improved healthcare access, medical advancements, and better lifestyle choices. However, between 2019 and 2020, the increase slowed significantly, with LEX rising only marginally from 77.968 to 78.077 years. This stagnation may be linked to the COVID-19 pandemic, which temporarily impacted global health systems and increased mortality rates. China has remarkably improved healthcare and living conditions over the past six decades. Although the rate of increase has slowed in recent years, the overall trend remains positive, reflecting continuous advancements in medical care and public health initiatives.

The primary objective of this study is to comprehensively examine the impact of various economic and social determinants on life expectancy (LEX) in China. Economic factors considered in this analysis include GDP, inflation, and health expenditure, while social factors encompass birth rate, EDU, population growth, and mortality rate. These variables collectively provide a holistic understanding of China's key drivers influencing life expectancy. To the best of the authors' knowledge, no prior research has investigated this specific set of economic and social regressors in the context of China. This study, therefore, makes a valuable contribution to the existing literature in two significant ways. First, it integrates a comprehensive set of economic and social determinants to examine their combined effects on life expectancy, which has remained unexplored in previous studies. Second, by analyzing these determinants, the study aims to enhance policy understanding related to sustainable development, particularly regarding health sustainability in China. The empirical findings from this study are expected to offer valuable insights for policymakers and management authorities, aiding them in devising effective strategies to improve public health outcomes. By identifying the key economic and social drivers of life expectancy, this research provides a foundation for informed decision-making to ensure long-term improvements in healthcare accessibility, economic stability, and social wellbeing in China.

The rest of this article is structured as follows: the “Review of Literature” section provides an overview of relevant theoretical and empirical studies. The “Data Description, Model Construction, and Empirical Methodology” section outlines the data and methodology used in the study. The “Discussion on Empirical Results” section interprets the findings. Finally, the “Conclusion and Policy Recommendations” section offers the study's conclusions.

## 2 Literature review

### 2.1 Economic determinants of life expectancy

#### 2.1.1 The relationship between economic growth/GDP and LEX

Economic growth is important for enhancing LEX as it enables better healthcare access, improved living standards, and stronger public health systems. Shahbaz et al. (37) analyzed the relationship between financial development, globalization, economic growth (EG), and LEX in Sub-Saharan African countries from 1970 to 2012 using non-linear and non-parametric econometric methods. The findings revealed a positive impact of financial development, globalization, and EG on LEX in most Sub-Saharan African economies, except in Gabon and Togo. Wang et al. (6) examined the dynamic relationship between EG and LEX in Pakistan from 1972 to 2017, with a focus on the roles of energy consumption and financial development. Using advanced unit root tests and the ARDL bounds testing technique. Their results indicated a positive association between EG and LEX. Wang et al. (38) explored whether renewable energy contributes to increase LEX. The study employed panel data from 121 countries and regions between 2002 and 2018 using a linear fixed-effect model and a non-linear panel threshold model. Their findings showed that the correlation between renewable energy consumption and LEX strengthens as GDP crosses specific thresholds. They recommended integrating health and renewable energy policies to maximize the health benefits of renewable energy. Nkalu and Edeme (7) investigated the relationship between environmental hazards and LEX in Africa using a GARCH model with data spanning from 1960 to 2017. The results indicated that income extended LEX by ~1.5 years, albeit with statistical insignificance. Segbefia et al. (1) studied the nexus between renewable energy, technological innovation, carbon emissions, and LEX in NAFTA economies from 1990 to 2020. Using the cross-sectional autoregressive distributed lag (CS-ARDL) model. The results highlighted that EG positively impacts LEX by improving healthcare access, living standards, and overall quality of life.

#### 2.1.2 The relationship between inflation (INF) and LEX

INF plays a crucial role in shaping LEX by influencing the affordability and accessibility of healthcare, nutrition, and essential services. Lawal et al. (4) investigated the relationship between INF and LEX in Nigeria using a Granger causality approach. They analyzed data from 1980 to 2019 with a Vector Auto Regression (VAR) model. The Granger causality results revealed no causal relationship between LEX and the inflation rate, indicating that inflation trends in Nigeria did not significantly influence LEX during the study period. Atia et al. (39) examined the factors affecting LEX in Bangladesh using data from 2000 to 2020. They applied multiple linear regression analysis. Their findings showed that both INF and unemployment were negatively associated with LEX in Bangladesh. They recommended controlling unemployment and inflation rates and increasing the number of physicians to improve LEX. Bao et al. (3) analyzed the nexus between real estate prices, inflation, and health outcomes in developed economies, including Canada, France, Japan, the Netherlands, Spain, Switzerland, Sweden, the United Kingdom, and the USA, from 1996 to 2019. They used fixed effects, random effects, two-stage least squares, and a generalized method of moments (GMM) for their analysis. Their results indicated that inflation positively affected infant mortality rates and negatively impacted LEX. Monsef and Mehrjardi (14) explored the determinants of LEX using a panel data approach across 136 countries from 2002 to 2010. They employed Generalized Least Squares and a conventional panel model with unobserved individual effects and time-varying coefficients. Their results demonstrated that both unemployment and inflation negatively affected LEX. Panahi and Aleemran (40) studied the effects of INF, health expenditure (HE), and urbanization on LEX in Middle Eastern and North African (MENA) countries from 2000 to 2012. They conducted a causal analysis. Their findings showed that a one-unit increase in inflation reduced LEX by 3.17 units, highlighting the negative impact of inflation.

#### 2.1.3 The relationship between health expenditure (HE) and LEX

HE plays a crucial role in improving public health outcomes by enhancing medical infrastructure, healthcare accessibility, and disease prevention efforts. Rahman et al. (41) explored the nexus between HE and health outcomes in 15 countries within the ASEAN region over 20 years (1995–2014). Using panel data analysis with fixed and random effect models. Their results showed that total, public, and private HE significantly reduced infant mortality rates. Additionally, private HE played a key role in reducing crude death rates. Setiawan et al. (42) examined the linkages between EG and LEX in Indonesia using time-series data from 1990 to 2021. Employing the Two-Stage Least Squares (2SLS) method for simultaneous equation modeling. Their findings revealed that healthcare expenditure and GDP per capita positively impacted LEX. Ullah et al. (15) analyzed the relationship between public HE and Pakistan's health outcomes from 1995 to 2017. Using the Quantile Autoregressive Distributed Lag (QARDL) approach. Their results showed that public HE significantly improved health outcomes, increasing LEX while reducing death rates and infant mortality in the short and long term. Radmehr and Adebayo (10) investigated the impact of HE on LEX in Mediterranean countries from 2000 to 2018. Applying the Method of Moments Quantile Regression (MMQR) approach. Their findings indicated that HE positively influenced LEX across all quantiles (0.1–0.90). Liang and Tussing (65) studied the cyclicality of government HE and its effects on population health using data from 135 developing countries from 1995 to 2014. Employing the Two-Stage Least Squares method. Their results revealed that reducing the procyclicality of government HE led to significant health improvements, with a one-unit cyclical reduction corresponding to ~0.97 additional life years per person and 13.1 lives saved per 1,000 adults.

### 2.2 Social determinants of life expectancy

#### 2.2.1 The relationship between population growth (PG) and LEX

PG influences LEX by affecting healthcare resources, economic development, and environmental conditions. Rapid PG can strain health systems, while controlled growth allows for better healthcare access and improved living standards, leading to higher LEX. Popoola (33) investigated the nexus between PG and LEX in Nigeria using time-series data from 1986 to 2015. Employing Granger Causality tests and regression analysis. The results indicated that rising PG positively, though insignificantly, impacted LEX. Additionally, a 1% decrease in fertility rate and the dependency ratio for those aged 65 and above could significantly improve longevity by 5.84 and 81.5, respectively. Granger causality tests revealed that population growth could drive low LEX at a 10% significance level. Hasnawati et al. (35) analyzed the relationship between LEX, PG, carbon dioxide emissions, and GDP growth in Indonesia from 1950 to 2020. Using a Vector Error Correction Model and Granger–Causality tests, they found that LEX was bidirectionally related to population growth. EG, measured by GDP, significantly influenced LEX, while carbon dioxide emissions impacted population growth but did not directly affect LEX or GDP. Felix and Onyenze (43) explored the nexus between PG, literacy, and LEX in Nigeria using secondary data from 1981 to 2018. They employed the Toda-Yamamoto non-Granger causality test based on an augmented VAR model. Results showed that population growth positively and significantly influenced LEX, with a bidirectional causality between LEX and population growth. Ayano (30) studied the impacts of PG dynamics on health in the Nigerian economy using time-series data from 1980 to 2018. They employed Ordinary Least Squares for estimation. Their findings revealed a negative and insignificant relationship between population and LEX. They recommended implementing strategies for effective population management, including family planning and educational programs, to ensure population dynamics positively impact health outcomes.

#### 2.2.2 The relationship between education (EDU) and LEX

EDU positively impacts LEX by promoting health awareness, disease prevention, and better lifestyle choices. Danler and Pfaff (27) investigated the impact of educational inequality on LEX disparities across 31 European countries between 1970 and 2010. Using linear regression models, they found that educational inequality was significantly associated with inequalities in LEX. Their results indicated that as educational disparities increase, so do disparities in longevity. The study also revealed that confounding factors, such as health behaviors, did not have a statistically significant separate effect on LEX inequality. Iyakaremye and Tripathi (18) analyzed the impact of EDU on LEX in Rwanda using data from the World Bank (1965–2020) and employed causal-comparative approaches, including cointegration tests and vector error correction models. Their results showed that education positively influences fertility rates, with higher levels of education leading to lower fertility. LEX increases across all educational groups, although gender disparities persist, which suggests growing uncertainty over time. Using cointegration and causality tests, Moga Rogoz et al. (29) examined the impact of economic freedom and educational attainment on LEX in new EU Member States during 2000–2019. Their findings revealed that economic freedom and EDU attainment significantly influence LEX in the short and long term. However, EDU attainment substantially affected LEX more than economic freedom. Gunu (44) explored the nexus between EDU and LEX in Ghana, analyzing the impact of educational policies using annual data from the World Bank (1992–2017) and employing ordinary least squares (OLS) techniques. The study found that EDU positively and significantly impacted LEX in Ghana. Government spending on EDU directly improved individuals' health and EG, positively influencing LEX. Luy et al. (45) examined the impact of rising EDU levels on LEX in Italy, Denmark, and the USA from 1990 to 2010, using a decomposition analysis with the replacement decomposition technique. Their results indicated that higher EDU levels significantly contributed to rising LEX by influencing mortality rates and population structure.

#### 2.2.3 The relationship between mortality rate (MR) and LEX

MR and LEX are inversely related, as a higher MR indicates shorter lifespans and poorer health conditions. Baran et al. (46) analyzed the dynamics of mortality and LEX among the working-age population in Kuzbass, Russia, from 2011 to 2018. Utilizing statistical data, short mortality tables based on age-specific mortality rates, and graphical analysis techniques. Their findings revealed that 2018 mortality rates in specific age groups surpassed those of 2011, negatively affecting LEX. LEX decreased by 0.57 years for men and 0.41 years for women in rural areas due to increased mortality, while urban areas experienced only minor losses. Azam et al. (47) explored the determinants of LEX in Pakistan using data from 1975 to 2020. They applied the ARDL bounds testing approach. The long-run results indicated that the death rate negatively affects LEX, while per capita income, urbanization, PG, birth rate, infant mortality rate, HE, and education positively impact LEX. Mourad (48) investigated the impact of socio-economic variables on LEX in 138 countries for the year 2010. Using Weighted Generalized Least Squares and multiple linear regression. The findings showed that an increase in child mortality significantly reduces LEX, with each additional deceased child lowering LEX by about 2.12 months. HE positively influenced LEX, with a $100 increase in per capita, extending LEX by ~33 days.

#### 2.2.4 The relationship between birth rate (BR) and LEX

BR significantly impacts LEX by influencing population dynamics, healthcare demand, and resource allocation. Zhang et al. (49) examined the relationship between the number of births and women's biological aging, premature mortality, and LEX in a prospective cohort study using data from the UK Biobank (2006–2010). The study involved 272,494 participants who completed a questionnaire regarding their number of live births. The researchers employed general linear regression models and Cox proportional hazards models to assess the effects of birth rate on women's biological aging, premature mortality, and LEX. The findings revealed a U-shaped association, where women with no children or five or more children experienced accelerated aging, higher risks of premature death, and lower LEX. Conversely, women with two children showed slower aging, a lower risk of premature death, and a higher LEX. Ayano (30) explored the effects of population dynamics on health in Nigeria using secondary time series data from 1980 to 2018 and applied Ordinary Least Squares (OLS) for analysis. The results indicated a negative and insignificant relationship between birth rate and LEX, with a coefficient of −0.01233, suggesting that a 100% increase in the birth rate could decrease LEX by 1.2%. Sabra (2022) examined the nexus between HE, LEX, fertility rate, CO2 emissions, and EG in six middle-income MENA countries from 2000 to 2019. Using Dynamic Panel Data system analysis, the study found a negative relationship between fertility rate and LEX, indicating that higher fertility rates were associated with lower LEX.

### 2.3 Research gap

Despite extensive research on the determinants of LEX, a significant gap remains in the literature regarding the simultaneous examination of economic and social factors influencing LEX in China. Most existing studies focus on economic determinants (such as GDP and health expenditure) or social determinants (such as education and mortality rate) in isolation rather than considering their combined effects on LEX. This fragmented approach limits a comprehensive understanding of the key drivers that contribute to improving public health and longevity in China. Moreover, while previous research has examined individual determinants of LEX in different regions, there is a lack of a unified framework that integrates multiple economic and social factors specifically for China. Given China's rapid economic growth, demographic transitions, and evolving healthcare policies, understanding how these factors influence LEX is crucial. However, no prior study has systematically analyzed this particular set of regressors within the Chinese context, leaving an important gap in the literature. Another critical research gap pertains to the policy implications of LEX determinants in the context of sustainable development. While several studies have explored life expectancy trends, few have explicitly linked these findings to policy recommendations for long-term health sustainability in China. The need for evidence-based policymaking is particularly relevant given China's commitment to improving public health as part of its broader sustainable development goals (SDGs). This study aims to bridge this gap by providing a deeper analysis of LEX economic and social drivers and their implications for healthcare policy and sustainability in China.

## 3 Methodology and data

### 3.1 Model specification

This study explores the economic and social factors influencing life expectancy. The model is based on previous literature, including Azam et al. (47), Ţarcă et al. (50), Rahman et al. (51).


(1) $$\begin{array}{l} LEX_{t}=\emptyset_{0}+\emptyset_{1}GDP_{t}+\emptyset_{2}INF_{t}+\emptyset_{3}HE_{t}+\emptyset_{4}PG_{t}\\ \;+\;\emptyset_{5}BR_{t}+\emptyset_{6}EDU_{t}+\emptyset_{7}MR_{t}+e_{t} \end{array}$$

In Equation 1, GDP, INF, HE, PG, BR, EDU, and MR represent the life expectancy, gross domestic product, inflation, health expenditure, population growth, birth rate, education and mortality rate, respectively. The term ∅_0_ and ∅_1_ *to* ∅_7_ signifies the intercept and slope of the respective independent variables.

### 3.2 Estimations strategy

The Augmented Dickey–Fuller (ADF) test was developed by Dickey and Fuller (52) to test for the presence of a unit root in a time series. The test extends the basic Dickey–Fuller test by including lagged differences of the dependent variable to account for higher-order autocorrelation. The equation for the ADF test is:


$$\Delta Y_{t}=\alpha +\text{β}t+\gamma Y_{t-1}+\sum_{i=1}^{p}\delta_{i}\Delta Y_{t-i}+\epsilon_{t}$$

This model tests the presence of a unit root in the time series by examining the relationship between the current change in the series (Δ*Y*_*t*_) and its past values, including a constant term (α), a time trend (β*t*), and lagged differences ($\overset{p}{\underset{i=1}{\sum }}\delta_{i}\Delta Y_{t-i}$) to control for autocorrelation. The test checks whether the coefficient of the lagged value (γ) is significantly different from zero to determine if the series is stationary (53).

In this study we use the Johansen and Juselius (54) Cointegration test. This test is a method used to detect Cointegration among multiple time series, identifying the number of cointegrating relationships through maximum likelihood estimation. It employs two statistics: the trace test and the maximum eigenvalue test, to assess the presence of cointegration. This test is commonly applied in econometrics to examine long-term relationships between variables.

An Autoregressive Distributed Lag (ARDL) model, developed by Pesaran and Shin (55), examines the relationship between a dependent variable and its own lags as well as the lags of independent variables. It is suitable for analyzing both short-term dynamics and long-term equilibrium relationships, even with variables of mixed integration orders *I*(0) and *I*(1). The ARDL model is widely used in econometrics due to its flexibility in handling small sample sizes and avoiding pre-testing for unit roots. According to Kong et al. (56) the ARDL model has calculated in two steps:

In the first step, the ARDL model is used to test for the presence of a long-term causal relationship between the variables by establishing the specified model.


(2) $$\begin{array}{l} \Delta LEX_{t}=\beta_{0}+\beta_{1}LEX_{t-1}+\beta_{2}GDP_{t-1}+\beta_{3}INF_{t-1}\\ \;+\;\beta_{4}HE_{t-1}+\beta_{5}PG_{t-1}+\beta_{6}BR_{t-1}+\beta_{7}EDU_{t-1}\\ \;+\;\beta_{8}MR_{t-1}\\ \;+\;\sum_{i=1}^{a}\beta_{5,i}\Delta LEX_{t-i}+\sum_{i=0}^{b}\beta_{6,i}\Delta GDINF_{t-i}P_{t-i}\\ \;+\;\sum_{i=0}^{c}\beta_{7,i}\Delta +\;\sum_{i=0}^{d}\beta_{8,i}\Delta HE_{t-i}+\sum_{i=0}^{e}\beta_{9,i}\Delta PG_{t-i}\\ \;+\;\sum_{i=0}^{f}\beta_{10,i}\Delta BR_{t-i}+\sum_{i=0}^{g}\beta_{11,i}\Delta EDU_{t-i}\\ \;+\;\sum_{i=0}^{h}\beta_{12,i}\Delta MR_{t-i}+u_{t} \end{array}$$

The ARDL model incorporates the first-order differential operator Δ to capture short-term dynamics, with *u*_*t*_ representing white noise. The maximum lag orders a, b, c, d, e, f, g, and h are selected using criteria such as AIC or BIC. The existence of a long-term equilibrium relationship among the variables is tested using the *F-*statistic, where the null hypothesis asserts no long-term equilibrium relationship.

In the second step, the ARDL model is used to analyze both long-term and short-term relationships between the variables, with the long-term relationship estimated through the ARDL (*p*_1_, *to p*_8_) specification.


(3) $$\begin{array}{l} LEX_{t}=\emptyset_{0}+\sum_{i=1}^{p_{1}}\emptyset_{1}LEX_{t-1}+\sum_{i=1}^{p_{2}}\emptyset_{1}GDP_{t-1}+\sum_{i=1}^{p_{3}}\emptyset_{1}INF_{t-1}\\ \;+\;\sum_{i=1}^{p_{4}}\emptyset_{1}HE_{t-1}+\sum_{i=1}^{p_{5}}\emptyset_{1}PG_{t-1}+\sum_{i=1}^{p_{6}}\emptyset_{1}BR_{t-1}\\ \;+\;\sum_{i=1}^{p_{7}}\emptyset_{1}EDU_{t-1}+\sum_{i=1}^{p_{8}}\emptyset_{1}MR_{t-1}+\varepsilon_{i} \end{array}$$

The short-term relationship can be analyzed using the ARDL-ECM model, as outlined in Equation 4.


(4) $$\begin{array}{l} \Delta LEX_{t}=\gamma_{0}+\sum_{i=1}^{a}\beta_{1}\Delta LEX_{t-i}+\sum_{i=0}^{b}\beta_{2}\Delta GDINE_{t-i}\;\\ \;+\sum_{i=0}^{c}\beta_{4}\Delta HE_{t-i}+\sum_{i=0}^{d}\beta_{5}\Delta PG_{t-i}+\sum_{i=0}^{e}\beta_{6}\Delta BR_{t-i}\\ \;+\;\sum_{i=0}^{f}\beta_{7}\Delta EDU_{t-i}+\sum_{i=0}^{g}\beta_{8}\Delta MR_{t-i}+\\ \;+\;\delta_{0}ECM_{t-1}+u_{t} \end{array}$$

Following the ARDL estimation, this study will employ Fully Modified Ordinary Least Squares (FMOLS) and Dynamic Ordinary Least Squares (DOLS) for robustness analysis. These methods are designed to estimate cointegrating relationships while addressing issues of endogeneity and serial correlation. FMOLS, developed by Phillips and Hansen (57), enhances the Ordinary Least Squares (OLS) approach by adjusting for autocorrelation in residuals and the endogeneity of regressors, ensuring more reliable long-run estimates. Meanwhile, DOLS, introduced by Stock and Watson (58), improves estimation accuracy by incorporating leads and lags of the first differences of the regressors, effectively mitigating both endogeneity and serial correlation. Both FMOLS and DOLS provide consistent and efficient estimates in cointegrated systems, strengthening the robustness of the empirical findings in this study (59).

### 3.3 Data and variables

This study examines the impact of economic and social determinants of LEX in china. The data has been obtained from WDI and UNDP from 2000 to 2023. Table 1 shows the data sources. We convert LEX, GDP, BR, EDU, and MR into natural logarithms except INF, HE and PG because it is already in the percentage form. Converting data into natural logarithms helps stabilize variance, reducing issues like Heteroscedasticity and data sharpness. It also linearizes relationships and allows regression coefficients to be interpreted as elasticities, improving model interpretability (60).

**Table 1:** Variable measurement.

**Symbols**	**Variables**	**Measurements**	**Sources**
LEX	Life expectancy	at birth, total (years)	WDI
GDP	Gross domestic product	GDP per capita (constant 2015 US$)	
INF	Inflation	Annual %	
HE	Health expenditure	% of GDP	
PG	Population growth	Annual %	
BR	Birth rate	Crude (per 1,000 people)	
EDU	Education	Mean Years of Schooling	UNDP
MR	Mortality rate	Infant (per 1,000 live births)	WDI

## 4 Results and discussions

### 4.1 Descriptive statistics

Table 2 shows the mean value of LEX, GDP, INF, HE, PG, BR, EDU, and MR are 4.328, 8.660, 2.093, 4.653, 0.495, 11.756, 13.061, and 13.281, respectively. While the maximum value of LEX, GDP, INF, HE, PG, BR, EDU, and MR are 4.364, 9.407, 5.925, 5.594, 0.788, 14.570, 15.218, and 29.900, respectively. The standard deviation of LEX, GDP, INF, HE, PG, BR, EDU, and MR are 0.026, 0.549, 1.708, 0.533, 0.227, 2.126, 1.711, and 7.763, respectively.

**Table 2:** Descriptive statistics.

**Variable**	**Mean**	**Median**	**Maximum**	**Minimum**	**Std. dev**.	**Skewness**	**Kurtosis**	**Jarque–Bera**	***p*-value**
LEX	4.328	4.331	4.364	4.275	0.026	−0.373	2.010	1.536	0.464
GDP	8.660	8.759	9.407	7.693	0.549	−0.340	1.811	1.875	0.392
INF	2.093	1.948	5.925	−0.732	1.708	0.563	3.091	1.275	0.529
HE	4.653	4.537	5.594	3.675	0.533	0.104	1.962	1.121	0.571
PG	0.495	0.566	0.788	−0.100	0.227	−1.390	4.083	8.906	0.012
BR	11.756	12.215	14.570	6.770	2.126	−1.140	3.300	5.291	0.071
EDU	13.061	13.221	15.218	9.905	1.711	−0.356	1.936	1.640	0.440
MR	13.281	11.000	29.900	4.800	7.763	0.751	2.352	2.676	0.262

### 4.2 Unit root test and cointegration test results

Table 3 presents the results of the ADF test. Under the condition “With Constant,” the LEX, GDP, and INF variables are stationary, while HE, PG, BR, EDU, and MR variables are non-stationary. Under the conditions “With Constant & Trend,” INF and MR variables are stationary at level. After taking the first difference, all variables become stationary, indicating integration of order one *I*(1). The PP test is also shown in Table 3; under the condition “With Constant,” the LEX and INF are stationary at level, while the rest of the variables are non-stationary at level; after the first difference, all variables become stationary. Table 4 summarizes the results of the Johansen Cointegration. It confirms the presence of five Cointegrating equations based on Trace and Max–Eigen statistics, indicating multiple long-run relationships among the variables. Similarly, Table 5 shows the ARDL Bound test, the *F-*statistic of 7.20 exceeds the upper bound critical values at the 10%, 5%, and 1% significance levels (3.52, 4.01, and 5.06, respectively). This indicates that the null hypothesis of no Cointegration can be rejected. Therefore, there is evidence of a long-run relationship between the variables.

**Table 3:** ADF and PP test results.

**Unit root test table (ADF)**									
		**LEX**	**GDP**	**INF**	**HE**	**PG**	**BR**	**EDU**	**MR**
**At level**									
With Constant	*t*-Statistic	−3.262^*^	−2.674^***^	−3.871^*^	−0.066	−0.419	0.859	−0.944	−2.406
With Constant & Trend	*t*-Statistic	−1.286	1.232	−4.196^**^	−1.431	−1.790	−0.179	−2.089	−3.556^***^
**At first difference**									
With Constant	*t*-Statistic	−4.024^*^	−1.010	−5.268^*^	−4.623^*^	−4.327^*^	−4.636^*^	−5.877^*^	−3.256^**^
With Constant & Trend	*t*-Statistic	−5.980^*^	−3.704^**^	−5.094^*^	−4.828^*^	−2.349	−4.828^*^	−5.752^*^	−2.835
**Unit root test table (PP)**									
**At level**									
With Constant	*t*-Statistic	−10.093^*^	−1.814	−6.348^*^	−0.122	−0.258	0.655	−0.878	1.067
With Constant & Trend	*t*-Statistic	−0.521	0.203	−8.035^*^	−1.408	−1.302	−0.494	−2.089	−3.706^**^
**At first difference**									
With Constant	*t*-Statistic	−4.024^*^	−1.756	−6.982^*^	−4.608^*^	−2.297	−4.707^*^	−5.882^*^	−2.845^**^
With Constant & Trend	*t*-Statistic	−8.028^*^	−2.922	−6.818^*^	−4.835^*^	−3.306	−4.882^*^	−5.757^*^	−1.283

^*^, ^**^, and ^***^ represent 1%, 5%, and 10% level of significance.

**Table 4:** Results of Johansen cointegration test.

**Null hypothesis (H0)**	**Eigen values**	**Trace values**		**Max–Eigen values**	
		**Statistics**	**Prob**	**Statistics**	**Prob**
None	0.985	403.831^*^	0.000	108.534^*^	0.000
At most 1	0.981	295.297^*^	0.000	102.455^*^	0.000
At most 2	0.908	192.842^*^	0.000	62.122^*^	0.000
At most 3	0.897	130.720^*^	0.000	59.058^*^	0.000
At most 4	0.773	71.662^*^	0.000	38.545^*^	0.001
At most 5	0.558	33.117^**^	0.020	21.201^**^	0.049
At most 6	0.367	11.916	0.161	11.900	0.115
At most 7	0.001	0.016	0.899	0.016	0.899

^*^, ^**^, and ^***^ represent 1%, 5%, and 10% level of significance.

**Table 5:** Results of ARDL bound test.

**Test statistic**	**Value**	**Critical value**		
		**Significance**	**I(0), Lower bound**	**I(1), Upper bound**
F-statistic	7.20^*^	10%	2.45	3.52
		5%	2.86	4.01
		1%	3.4	5.06

^*^, ^**^, and ^***^ represent 1%, 5%, and 10% level of significance.

### 4.3 ARDL long run and short run results

Table 6 shows the ARDL estimates. In the long run, GDP, HE, BR, and EDU positively affect LEX, while INF, PG, and MR negatively affect LEX in China from 2000 to 2023. It accepts the alternative hypothesis. In the long run, the coefficient of GDP is positive; a 1% increase in GDP raises LEX by 0.31%, indicating that economic growth contributes positively to health outcomes. The reason for the positive is that higher GDP raises LEX by improving access to quality healthcare, better nutrition, and better living standards. Economic growth enables investments in public health infrastructure and Education, leading to healthier and longer lives. The finding is consistent with the finding of Shahbaz et al. (37) and Wang et al. (6). Shahbaz et al. (37) analyzed the relationship between EG and LEX in SSA economies, and they found that EG positively affects LEX. Wang et al. (6) examined the dynamic relationship between EG and LEX in Pakistan from 1972 to 2017; their results indicated a positive association between EG and LEX.

**Table 6:** The ARDL estimates.

**Variable**	**Coefficient**	**Std. error**	***t*-statistic**	**Prob**.
**Long run**				
GDP	0.312^***^	0.144	2.167	0.051
INF	−0.069^*^	0.021	−3.286	0.000
HE	0.156^*^	0.025	6.240	0.000
PG	−0.287^**^	0.101	−2.842	0.024
BR	0.168^*^	0.033	5.091	0.000
EDU	0.784^**^	0.312	2.513	0.039
MR	−0.245^*^	0.076	−3.224	0.000
**Short run**				
GDP	0.005	0.019	0.268	0.793
INF	−0.026^***^	0.012	−2.157	0.052
HE	0.012^*^	0.002	5.374	0.000
PG	−0.089^*^	0.008	−11.632	0.000
BR	−0.005	0.006	−0.794	0.442
EDU	0.038^**^	0.016	2.432	0.032
MR	−0.012	0.018	−0.657	0.524
ECM(−1)	−0.137^*^	0.029	−4.762	0.001

^*^, ^**^, and ^***^ represent 1%, 5%, and 10% level of significance. Dependent variable: LEX.

The coefficient of INF is negative; a 1% increase in inflation reduces LEX by 0.069%, showing that economic instability negatively affects health. Inflation reduces LEX by eroding purchasing power, making essential goods like food, medicine, and healthcare less affordable for individuals, especially in low-income households. High INF often leads to economic instability, unemployment, and reduced government spending on public health services. These factors worsen living conditions and access to healthcare, leading to poorer health outcomes and shorter lifespans. The finding is consistent with Atia et al. (39) and Bao et al. (3). Atia et al. (39) examined the factors affecting LEX in Bangladesh, and their findings showed that both INFs were negatively associated with LEX. Bao et al. (3) analyzed the nexus between inflation and health outcomes in developed economies; their results indicated that inflation positively affected infant mortality rates and negatively impacted LEX.

The coefficient of HE is positive, 1% increase in health expenditure boosts LEX by 0.156%, showcasing the importance of investing in healthcare. Thus, Health expenditure increases life expectancy by improving access to quality healthcare services, advanced medical treatments, and preventive care. It also strengthens public health infrastructure, leading to better disease management and healthier populations. The results are the same as the line of Rahman et al. (41), Setiawan et al. (42), and Radmehr and Adebayo (10). Rahman et al. (41) explored the nexus between HE and health outcomes in 15 countries and they found that total, public, and private HE significantly reduced infant mortality rates. Additionally, private HE played a key role in reducing crude death rates. Setiawan et al. (42) examined the linkages between EG and LEX in Indonesia. Their findings revealed that healthcare expenditure and GDP per capita positively impacted LEX. Radmehr and Adebayo (10) investigated the impact of HE on LEX in Mediterranean countries. Their findings indicated that HE positively influenced LEX across all quantiles. Ijaz (61) found that Benazir Income Support Programme (BISP) raised the health outcomes.

The coefficient of PG is negative, a 1% uptick in population growth (PG) lowers life expectancy by 0.287%, pointing to the challenges posed by resource constraints in rapidly growing populations. Thus Population growth negatively impacts life expectancy by straining resources like healthcare, Education, and housing, reducing their availability and quality. Overcrowding and environmental degradation further exacerbate health challenges, leading to poorer outcomes. The results are consistent with the line of Ayano (30) and Uddin et al. (60). Ayano (30) studied the impacts of PG dynamics on health in the Nigerian economy. Their findings revealed a negative and insignificant relationship between population and LEX. Uddin et al. (60) found that population growth reduced the life expectancy in south Asian economies.

The coefficient of BR is positive; a 1% increase in the birth rate raises life expectancy by 0.168%, potentially reflecting improved maternal and infant healthcare. A higher birth rate can positively affect LEX by reflecting maternal and infant healthcare improvements, leading to better newborn survival rates. It also indicates greater investments in public health systems that promote overall population wellbeing. This finding is consistent with the finding of Azam et al. (47), while contradict from the finding of Uddin et al. (60).

The coefficient of Education has a positive effect on LEX; a 1% growth in EDU leads to a 0.784% increase in life expectancy, underscoring the significant role of Education in improving health. Thus, Education improves life expectancy by increasing awareness of healthy lifestyles, disease prevention, and access to healthcare. It also contributes to economic stability and better living conditions, which promote longer and healthier lives. This finding is the same as the findings of Iyakaremye and Tripathi (18) and Moga Rogoz et al. (29). Iyakaremye and Tripathi (18) analyzed the impact of EDU on LEX in Rwanda. Their results showed that Education positively influences fertility rates, with higher levels of Education leading to lower fertility. LEX increases across all educational groups, although gender disparities persist, which suggests growing uncertainty over time. Moga Rogoz et al. (29) examined the impact of educational attainment on LEX in new EU Member States. Their findings revealed that EDU attainment significantly influences LEX in the short and long term. However, EDU attainment had a stronger effect on LEX than economic freedom.

The coefficient of MR is negative, indicating that a 1% rise in the mortality rate (MR) reduces life expectancy by 0.245%, reaffirming the negative effects of higher mortality rates. Higher mortality rates directly lower life expectancy by increasing the number of deaths in a population, particularly among vulnerable groups. This reflects poor health conditions, inadequate healthcare, and higher risks of disease and accidents. This outcome is the same outcome of Baran et al. (46). Baran et al. (46) analyzed the dynamics of mortality and LEX among the working-age population in Kuzbass. Their findings revealed that 2018 mortality rates in specific age groups surpassed those of 2011, negatively affecting LEX. LEX decreased by 0.57 years for men and 0.41 years for women in rural areas due to increased mortality, while urban areas experienced only minor losses.

The short-run ARDL estimates show that INF, PG, BR, and MR negatively affect LEX, while GDP, HE, and EDU positively affect LEX in China. The ECM(−1) = −0.137 coefficient in the life expectancy model indicates that any short-term deviation from the long-run equilibrium is corrected by 13.7% in each period. This negative sign suggests that the system adjusts toward equilibrium if life expectancy deviates from its long-term trend. The statistical significance of this term (indicated by the asterisk) shows that this adjustment process is reliable and significant.

### 4.4 ARDL diagnostic and robustness analysis

**Figure 1:**
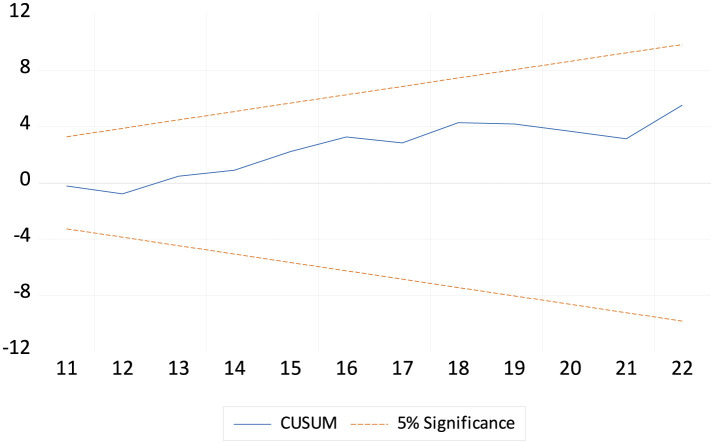
The cumulative sum of the recursive residual plot.

**Figure 2:**
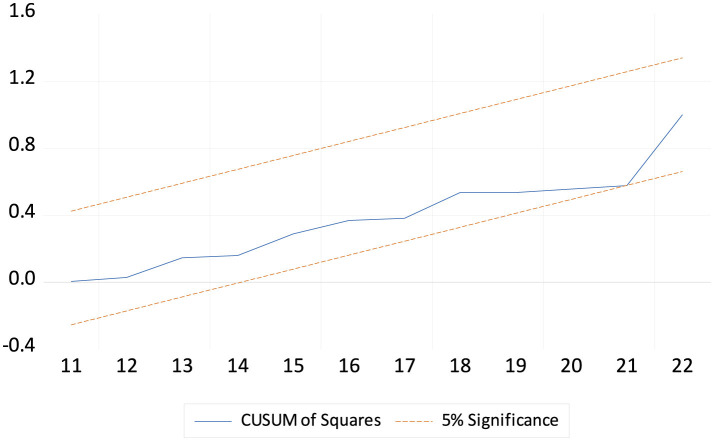
The cumulative sum of the square of the recursive residual plot.

Table 7 shows the ARDL diagnostic test estimates. The diagnostic tests for the life expectancy model show that the residuals are normally distributed (Jarque-Bera test *p-*value = 0.875), and there is no significant autocorrelation (LM test *p-*value = 0.702). There is also no evidence of heteroscedasticity as indicated by the ARCH (*p*-value = 0.542) and BPG-LM tests (*p*-value = 0.679). The Ramsey RESET test (*p*-value = 0.208) also suggests that the model is correctly specified and stable. Overall, these results indicate that the model is robust and appropriately specified, with no significant residual or stability issues. Both Figures 1, 2 reported that LEX is stable. Table 8 shows the robustness analysis. The FMOLS and DOLS methods yield similar results to the ARDL model for life expectancy (LEX) in China from 2000 to 2023. Both methods confirm that GDP, HE, BR, and EDU positively impact LEX, indicating that economic growth, health expenditure, birth rate, and education contribute to improved life expectancy. Conversely, INF, PG, and MR negatively affect LEX, reflecting the adverse impact of inflation, population growth, and mortality rates on health outcomes. These consistent findings across the three methodologies highlight the robustness of the relationships between the variables and life expectancy.

**Table 7:** ARDL diagnostic analysis.

**Test**	***F*-stats**	**Prob**
Jarque–Bera test for normality	0.265	0.875
LM test for autocorrelation	1.051	0.702
ARCH test for heteroscedasticity	0.382	0.542
BPG-LM test for heteroscedasticity	0.779	0.679
Ramsey RESET test for stability	1.786	0.208

**Table 8:** Robustness analysis.

**Variable**	**FMOLS**			**DOLS**		
	**Coefficient**	**Std. error**	**Prob**.	**Coefficient**	**Std. error**	**Prob**.
GDP	0.319^*^	0.034	0.000	0.342	0.082	0.001
INF	−0.012^*^	0.001	0.000	−0.012	0.001	0.000
HE	0.030^*^	0.007	0.000	0.026	0.014	0.088
PG	−0.065	0.039	0.113	−0.128	0.175	0.477
BR	0.091^**^	0.040	0.034	0.462	0.190	0.021
EDU	0.213^**^	0.081	0.016	0.193	0.176	0.294
MR	−0.259^*^	0.044	0.000	−0.279	0.100	0.016
*R* ^2^	0.96			0.98		
Adj *R*^2^	0.94			0.96		

^*^, ^**^, and ^***^ represent 1%, 5%, and 10% level of significance. Dependent variable: LEX.

## 5 Conclusions and policy recommendations

The primary aim of this study was to examine the impact of economic and social determinants on LEX. The Economic factors include GDP, INF, and HE, while social factors include birth rate, EDU, population growth, and mortality rate. This study utilized the ARDL, FMOLS, and DOLS estimators from 2000 to 2023. The findings showed that GDP, HE, and EDU positively affect LEX, while population growth, birth rate, inflation, and mortality rate negatively affect LEX.

This empirical study offers several policy recommendations to enhance the LEX in China: First, The Chinese government should allocate funds toward improving healthcare facilities, ensuring wider access to quality medical services, and reducing mortality rates. Second, Increased funding for nutrition-focused initiatives can enhance food security, combat malnutrition, and improve overall health and LEX. Third, the government should use its growing economic resources to conduct public education campaigns on health and wellness, promoting preventive care and healthier lifestyles. Fourth, Stabilizing inflation is crucial to prevent rising healthcare costs, ensuring affordable access to medical services and essential medications. Fifth, Subsidizing essential goods like food and medicines during inflationary periods can help maintain proper nutrition and healthcare access, improving overall public health outcomes. Sixth, prioritizing health expenditure is necessary to expand access to hospitals, clinics, and preventive care, reducing disease prevalence and improving LEX. Seventh, Allocating health funds toward modern medical equipment and research enhances diagnostic accuracy and treatment effectiveness, leading to better health outcomes. Eight, Increased health spending should focus on training and retaining skilled medical personnel, ensuring high-quality healthcare delivery, and improving LEX. Ninth, Expanding healthcare infrastructure is essential to accommodate China's growing population, ensuring accessible and equitable medical care for all citizens. Ten, investing in community health initiatives such as vaccinations and awareness programs can help prevent diseases, directly enhancing public health and LEX. Eleven, Promoting education and family planning can help manage population growth sustainably, reducing resource strain and fostering healthier living conditions for longer lifespans. Twelve, strengthening maternal healthcare and neonatal facilities can significantly reduce mortality rates, directly contributing to higher LEX. Thirteen, Targeting significant causes of mortality, such as chronic diseases and epidemics, through preventive healthcare measures can enhance survival rates and boost LEX in China.

This study has several limitations that provide directions for future research. First, it focuses only on economic determinants of LEX, such as GDP, inflation, and health expenditure, while excluding other key economic factors like unemployment, per capita income, and income inequality, which may also influence LEX. Second, it considers only social determinants such as birth rate, education, population growth, and mortality rate, but does not include other crucial social factors like urbanization, environmental pollution, and healthcare accessibility, which could provide a more comprehensive understanding of LEX trends. Third, the study is limited to China's economy, restricting its applicability to other regions with different economic structures and healthcare systems. Future research should expand this analysis to both developing and developed economies, incorporating a wider set of economic and social determinants to provide more generalizable insights. This study employs the ARDL, FMOLS, and DOLS estimators. However, it does not incorporate the asymmetric ARDL model, which captures potential non-linearities in variable relationships. Furthermore, advanced econometric techniques such as structural break tests, threshold models, and regime-switching approaches are not included. The analysis remains limited to ARDL, FMOLS, and DOLS without delving into more complex time series methodologies.

## Data Availability

The raw data supporting the conclusions of this article will be made available by the authors, without undue reservation.
